# Enterobacterales and Antimicrobial Resistance in Feed, Water, and Slurry in Pig Production Farms in the Greater Accra Region of Ghana, 2024

**DOI:** 10.3390/tropicalmed10090239

**Published:** 2025-08-27

**Authors:** Elvis Fiam Amegayibor, Rita Ohene Larbi, Matilda Ayim-Akonor, Richael Odarkor Mills, Helena Owusu, Benjamin Kissi Sasu, Robert Fraser Terry, Anthony D. Harries, Florence S. Kuukyi

**Affiliations:** 1 Department of Biomedical Sciences, School of Allied Health Sciences, University of Cape Coast, PMB UCC Post Office, Cape Coast CC-123-1749, Ghana; 2 Council for Scientific and Industrial Research-Animal Research Institute, Achimota-Accra P.O. Box AH 20, Ghana; 3 Pharmacy Department, Korle Bu Teaching Hospital, Accra P.O. Box KB 77, Ghana; 4 AMR Reference Laboratory for Animal Health, National Food Safety Laboratory, Veterinary Services Directorate, Accra P.O. Box M 161, Ghana; 5 Special Programme for Research and Training in Tropical Diseases, World Health Organization, CH-1211 Geneva, Switzerland; 6 International Union Against Tuberculosis and Lung Disease, 75001 Paris, France; 7 Faculty of Infectious and Tropical Diseases, London School of Hygiene and Tropical Medicine, Keppel Street, London WC1E 7HT, UK; 8 Metro Public Health Department, Accra Metropolitan Assembly, Accra GP 385, Ghana

**Keywords:** West Africa, pig farms, *Escherichia coli*, *Enterobacter* spp., antimicrobial resistance (AMR), multi-drug resistance, phenotypic colistin resistance, operational research, SORT IT

## Abstract

Increasing antimicrobial resistance (AMR) levels in Enterobacterales from pigs in Ghana prompted us to investigate farm feed, pig slurry, and farm water for Enterobacterales isolates, their antimicrobial resistance patterns, and antimicrobial residues. Between August and November 2024, we collected one sample each of feed, slurry, and water from 14 pig farms for microbiological analysis. Out of 42 samples, Enterobacterales (*E. coli* and *Enterobacter* spp.) were isolated from 30 (71.4%) samples, with the highest prevalence found in feed (85.7%), followed by slurry (78.6%) and water (50.0%). The prevalence of AMR to tetracyclines, trimethoprim-sulfamethoxazole, and ampicillin was high, with over 50% of isolates from slurry and water and 40% from feed exhibiting tetracycline resistance. Multi-drug resistance (MDR) was identified in nine (27.3%) isolates of Enterobacterales, with the highest prevalence found in feed (38.5%), then slurry (23.1%), and water (14.3%). Among 42 farm samples screened for colistin-resistant Enterobacterales, 10 (23.8%) exhibited phenotypic colistin resistance. No antimicrobial residues were detected. Risk factors associated with MDR included large farms with high pig turnover (*p* < 0.05) and the channelling of slurry into both covered and uncovered pits on the farm (*p* < 0.05). These high resistance levels underscore the urgent need for improved hygiene in feed, water, and slurry management, stricter antibiotic stewardship with veterinary oversight, and better enforcement of existing antibiotic use regulations on pig farms.

## 1. Introduction

Antimicrobials are essential in modern livestock farming, where they are routinely used to prevent infections, treat diseases, and promote growth. Livestock, including pigs, are susceptible to various bacterial, viral, and parasitic infections. These infectious diseases can result in significant financial losses due to decreased productivity, increased treatment costs and higher mortality rates [1]. Farmers use antimicrobials as prophylactic measures to maintain the health and productivity of their animals. Additionally, when administered at subtherapeutic levels, antimicrobials can enhance growth rates by improving nutrient absorption, feed efficiency, and weight gain [2]. However, concerns arise from the overuse and misuse of antimicrobials, including the risk of promoting antimicrobial resistance (AMR) and environmental contamination.

Antimicrobials enter the environment through several routes, including wastewater discharges from households, hospitals, and pharmaceutical facilities, as well as agricultural runoff. This release of antimicrobial agents contributes to the selection pressure that drives bacterial evolution and the emergence of more resistant strains [3]. When natural bacterial communities come into direct contact with discharged resistant bacteria, the evolution of resistance is accelerated through the horizontal transfer of antimicrobial resistance genes (ARGs) [3].

The prevalence of AMR and multi-drug resistant (MDR) Gram-negative Enterobacterales has been increasing across Africa, particularly in West Africa, including Ghana [4,5,6]. A significant contributor to this public health threat is the increasing use and misuse of antimicrobial agents in both humans and animal husbandry [7].

MDR [defined as resistance to three or more antimicrobial classes] [8] is especially prevalent among Enterobacterales. In recent years, there has been a notable increase in MDR-Enterobacterales in animals, posing both a direct and indirect risk to public health [9]. Over the past 20 years, several studies in Africa have reported high levels of MDR Enterobacterales in pigs and pork meat [5,6,10], attributed to the widespread and increasing use of antimicrobials in animals for treatment and prophylaxis, as well as for growth promotion [11].

In this context, Ohene Larbi and colleagues conducted a study in 2022 on healthy pigs from commercial farms in the Greater Accra region of Ghana, focusing on Enterobacterales and their resistance profiles to antimicrobials commonly used in human and veterinary medicine [12]. The two primary bacteria identified in the pig rectal swabs were *Escherichia coli* (*E. coli*) and *Enterobacter* spp., with MDR prevalence at 23% and 5%, respectively. Phenotypic resistance to colistin was also found in 8% of both *E. coli* and *Enterobacter* spp. isolates, while molecular resistance (indicated by the presence of the *mcr-1* gene) was found in half of these isolates. Although all farms used antimicrobials for treatment and/or prophylaxis, farmers stated in the questionnaires that antimicrobials were not included in farm feed to promote growth. At the time of this study, Ghana’s regulatory policy on AMR made mention of the responsible use of antibiotics in animal husbandry. However, guidelines to ensure the implementation of this policy had not been developed. As such, one of the key recommendations of the study was to strengthen and ensure implementation of regulatory policy, especially on the use of colistin in animal husbandry.

Following the study, the findings were shared with various stakeholders, and several recommendations (such as the one made above) were made. One key recommendation, pertinent to this study, was to assess farm feeds and other farm samples, such as pig slurry and farm water, for Enterobacterales and their antimicrobial resistance patterns as well as for antimicrobial residues. This assessment aimed to determine if these samples contained active antimicrobial ingredients that could contribute to the spread of AMR and MDR. The aim of the study, therefore, was to assess farm feeds and environmental samples of pig slurry and farm water for antimicrobial resistance patterns (including colistin) of Enterobacterales (*E. coli* and *Enterobacter* spp.) and antimicrobial residues in 14 farms in the Greater Accra region of Ghana in 2024. Specific objectives included documenting: (i) farm characteristics and practices; (ii) Enterobacterales, their associated patterns of AMR, MDR, and resistance to colistin, and antimicrobial residues; and (iii) associations between farm characteristics and practices with MDR in Enterobacterales isolated from farm feed, pig slurry, and farm water.

## 2. Materials and Methods

### 2.1. Study Design

This cross-sectional study used primary data collected from pig farms.

### 2.2. General Setting

Ghana is situated along the Gulf of Guinea and the Atlantic Ocean of West Africa. As of 2021, Ghana’s population was about 30.8 million, and the Greater Accra region, which hosts the capital city Accra, accounted for 5.4 million [13].

### 2.3. Site Specific Setting

There are 29 districts that constitute the Greater Accra region of Ghana [13]. Using information from the Greater Accra Pig Farmers Association and the Ministry of Food and Agriculture (MOFA), four districts (Adenta, Ningo-Prampram, Shai-Osudoku, and Ga South) with a high number of commercial pig farms were selected. In the first study by Ohene Larbi et al. in 2022 [13], five pig farms were randomly selected within each district, making a total of twenty farms. In this current study in 2024, six of those farms in the four districts stopped pig production between 2022 and 2024, and thus, fourteen of those original twenty farms were assessed.

### 2.4. Study Population

The study population included three samples taken from each of the 14 selected farms in the months of August to November 2024: in total, 42 samples were from farm feed, pig slurry, and farm water.

### 2.5. Farm Visits and Sample Collection

Farm visits were carried out on a district-by-district basis. The study team consisted of a veterinarian, a field assistant, a Livestock Extension Officer of MOFA, and the principal investigator. At each of the 14 farms, the team explained to the farmers the purpose and details of the study. Once the farmer agreed to participate in the study, a consent form was given to the farmer to sign or thumbprint. In each farm, a short questionnaire was administered to assess farm characteristics and practices and antimicrobial use. Three samples were then taken: one sample from the farm feed, one sample from pig slurry, and one sample from the main source of animal drinking water (borehole, tap water, or well water). Farm feed (5 g) was collected into sterile zipped bags. Approximately 200 mL of each of the pig slurry and farm water was collected into sterile plastic containers. All the samples were kept on ice and transported to the microbiology laboratory of the Centre for Scientific and Industrial Research (CSIR)—Animal Research Institute, where they were processed within 12–48 h of collection.

### 2.6. Sample Processing and Microbiological Procedures

In the laboratory, farm feed and pig slurry samples were homogenized with deionized water to ensure uniform distribution. Each mixture was then vortexed. The agar well diffusion method was used to determine the presence of antimicrobial residues [14]. Wells were created aseptically in petri-plates inoculated with *Bacillus subtilis* using a sterile cork borer. A total of 100 µL of each homogenized sample mixture and each farm water sample (100 µL) were added to the wells. Positive and negative controls (standard antibiotic solutions and sterile water) in additional wells were created. The plates were then incubated at 37 °C for 24 h, at which point inhibition zones around each well were measured and compared with those of controls.

Each feed sample (1 g) was weighed into 9 mL of buffered peptone water for pre-enrichment and incubated overnight, after which a 10 µL loopful was cultured on MacConkey agar [15]. A loopful of pig slurry was plated onto MacConkey agar [Oxoid Ltd., Hampshire, UK] [16]. A total of 100 uL of each farm water sample was plated out on MacConkey agar by spread plating after centrifuging at 1500 rpm for 15 min [17]. These were incubated at 37 °C for 24 h. From each farm sample, we selected three colonies that showed distinct bacterial morphology of *E. coli* and *Enterobacter* spp. respectively. These were then streaked onto Eosin Methylene Blue agar (Oxoid Ltd., Hampshire, UK) and incubated under the same conditions. Standard biochemical tests (including Simmons Citrate, Triple Sugar Iron, and Indole) were performed to ensure further confirmation of these isolates. Antimicrobial susceptibility testing was carried out on confirmed *E. coli* and *Enterobacter* spp. isolates using the following antimicrobials: ceftazidime (30 µg), gentamicin (10 µg), ampicillin (10 µg), amoxicillin-clavulanic acid (30 µg), tetracycline (30 µg), ciprofloxacin (5 µg), trimethoprim-sulfamethoxazole (25 µg), chloramphenicol (30 µg), and aztreonam (30 µg), in line with Clinical Laboratory Standards Institute (CLSI) guidelines [18].

To screen for bacterial resistance to colistin, all samples (from farm feed, pig slurry, and farm water) were plated directly in 10 µL volumes on Chromagar COL-APSE. Pink and blue isolates representing presumptive *E. coli* and *Enterobacter* spp. were incubated for 24 h at 37 °C, after which suspected *E. coli* and *Enterobacter* spp. colonies were selected for further biochemical testing. All colistin-resistant isolates were analysed molecularly to assess for the presence of the *mcr-1* gene. The DNA of *E. coli*, which was phenotypically colistin-resistant, was extracted by the boiling method. To establish the presence of the *mcr-1* gene, the primer sequence CLR5-F (5′CGGTCAGTCCGTTTGTTC-3′) and CLR5-R (5′-CTTGGTCGGTCTGTA GGG-3′) was used for polymerase chain reactions (PCR) [5,6]. Total reaction volume was set at 25 μL by adding 5 μL of 2× master mix, 1 μL each of forward and reverse primer, 6 μL of DNA template, and 12 μL of double-distilled water. The optimized PCR amplification cycle of the *mcr-1* gene was 94 °C for 2 min; 40 cycles of denaturation at 94 °C for 1 min; annealing at 59 °C for 1 min; extension at 72 °C for 2 min, final extension at 72 °C for 10 min, and holding at 4 °C for infinity. Amplicons (with expected fragment size of 309 bp) were resolved on a 1.5% ethidium bromide-stained agarose gel and visualized with a UV transilluminator (Gel Doc).

### 2.7. Data Variables and Sources of Data

Data variables included: ID of pig farm; farm characteristics and practices; samples from each farm—farm feed, pig slurry, and main farm water source; antimicrobials used on the farms; antimicrobial residues; isolates of *E. coli* and *Enterobacter* spp.; for each *E. coli* and *Enterobacter* spp. isolate, antimicrobial susceptibility or resistance (defined as intermediate or resistant) to ceftazidime, gentamicin, ampicillin, amoxicillin-clavulanic acid, tetracycline, ciprofloxacin, trimethoprim-sulfamethoxazole, chloramphenicol, and aztreonam. MDR was documented if a bacterial isolate demonstrated resistance to three or more of the antimicrobial classes; in this definition, we excluded colistin. For colistin, the variables were phenotypically sensitive or resistant *E. coli* and *Enterobacter* spp., and genotypic resistance was reported based on the detection of the *mcr-1* gene.

### 2.8. Data Management, Analysis, and Statistics

Farm characteristics, Enterobacterales isolates in the three samples, their patterns of AMR/MDR and colistin-resistance, and antimicrobial residues were collected using the free mobile data-capture application called Epicollect5 [19]. Using ID numbers, samples and antimicrobial residues were linked to the specific farms and, in turn, these were linked to the CSIR Animal Research Institute Laboratory data. The Epicollect5 data were extracted and exported to Jamovi version 2.3.28, where further cleaning and analysis took place.

A descriptive analysis was performed to determine frequencies and proportions from categorical variables such as samples, isolates of *E. coli* and *Enterobacter* spp., their AMR, MDR, and phenotypic and genotypic colistin resistance patterns, and antimicrobial residues. Associations between farm characteristics and practices and MDR in Enterobacterales were assessed using the chi-squared test and presented as Prevalence Ratios (PRs) with 95% confidence intervals (CIs). Levels of significance were set at 5% (*p* < 0.05).

## 3. Results

### 3.1. Characteristics and Practices of Selected Pig Farms Including Their Antimicrobial Use, Farm Feed, Farm Water, and Waste Management

Farm characteristics and practices are shown in Table 1. The median (IQR) pig production turnover in the 14 farms was 165 (85–215) pigs per annum. Ten (71.4%) farms used self-made farm feed, three (21.4%) used a combination of commercial and self-made feed, and one (7.2%) used commercial feed. Ingredients for self-made farm feed were usually organic products, milled at a local feed mill, with some farms adding toxin binders to prevent mycotoxin infections. No antimicrobials were added to farm feed. Boreholes (*n* = 8, 57.1%) were the commonest water source, followed by tap water (*n* = 4, 28.6%) and hand-dug well water (*n* = 2, 14.3%). Waste management practices varied. With pig fecal waste management, seven (50%) farms dumped the waste on the farm for later collection by crop farmers. With pig slurry, nine (64.3%) farms channelled it freely into farm soil. Few farms (*n* = 3) had functional foot baths, while farmers used personal protective equipment on all farms that included footwear and dedicated farm clothing. Antimicrobial use was common: eight (57.1%) farms had administered antibiotics for treatment in the past year, but in only half of those farms were veterinarians consulted, with the remainder of farmers making their own decisions about the type and dosage of antimicrobial. In six (42.9%) farms, prophylactic antimicrobials had been given to the pigs in the previous 12 months. The antimicrobials used on the farms included streptomycin/gentamicin, penicillins, tetracyclines, sulphonamides, trimethoprim, tylosin/erythromycin, enrofloxacin, and colistin. In six (42.9%) farms, the antimicrobial containers were either buried or dumped on the farm after they had been used.

### 3.2. Prevalence of Enterobacterales with Patterns of AMR, MDR, and Colistin Resistance in Farm Feed, Pig Slurry, and Farm Water Samples

The prevalence of Enterobacterales (*E. coli* and *Enterobacter* spp.) in the samples is shown in Table 2. A total of thirty (71.4%) Enterobacterales isolates were obtained from the three sample types, with the highest burden in farm feed, followed by pig slurry and farm water. In terms of specific bacteria, farm feed had the highest Enterobacter spp. prevalence of 66.7.% while pig slurry had the highest *E. coli* prevalence of 81.8% with one feed sample and two slurry samples having both *E. coli* and *Enterobacter* spp. present.

**Figure 1:**
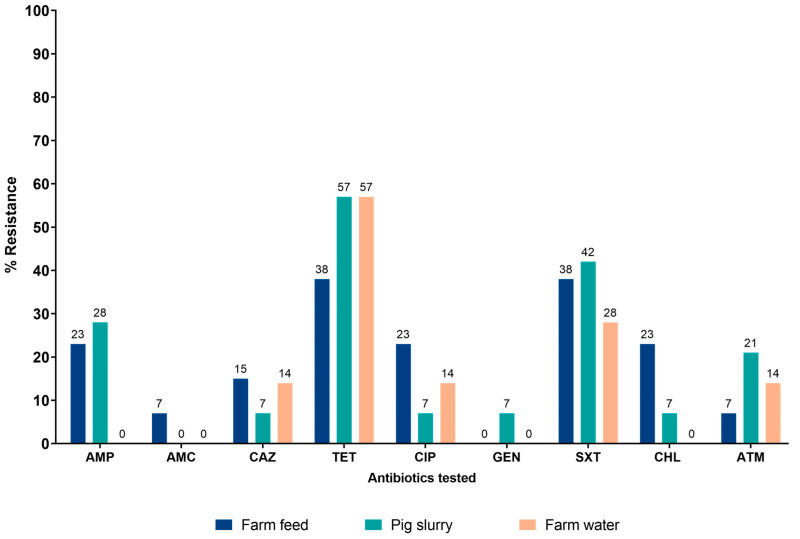
Percentage resistance of Enterobacterales from farm feed, farm water, and pig slurry. On selected farms in the Greater Accra region of Ghana, 2024. Footnote: AMP = Ampicillin; AMC = Amoxicillin clavulanic acid; CAZ = Ceftazidime; TET = Tetracycline; CIP = Ciprofloxacin; GEN = Gentamicin; SXT = Trimethoprim-sulfamethoxazole; CHL = chloramphenicol; ATM = Aztreonam.

The proportions of Enterobacterales isolated from farm feed, pig slurry, and farm water samples that were resistant to the selected antibiotics tested are shown in Figure 1. The most notable resistance was observed for tetracyclines, with over 50% of isolates from pig slurry and farm water, and nearly 40% of farm feed isolates being resistant. There was a high prevalence of resistance to trimethoprim-sulfamethoxazole, being just over and just under 40% in pig slurry and farm feed, respectively. Between 20 and 25% of Enterobacterales in pig slurry and farm feed were also resistant to ampicillin. In contrast, the lowest resistance was recorded for gentamicin, amoxicillin-clavulanic acid, and ceftazidime across all samples, with farm water showing the least resistance overall.

MDR was identified in 9 (27.3%) of the 33 Enterobacterales isolates. It was most prevalent in farm feed samples (38.5%), followed by pig slurry (23.1%) and farm water (14.3%). Most MDR isolates exhibited resistance to three antimicrobial classes (R3), with fewer showing resistance to four (R4) or five (R5) (Table 3).

Of the 42 farm samples screened for colistin-resistant Enterobacterales, 10 (23.8%) and 3 (7.1%) exhibited phenotypic and genotypic resistance (*mcr-1* gene), respectively (Table 4). *E. coli* accounted for the lowest proportion of phenotypic resistant isolates (3/10), while two-thirds showed genotypic resistance.

### 3.3. Antimicrobial Residues in Farm Feed, Pig Slurry, and Farm Water Samples

No antimicrobial residues were found in any of the samples tested on the 14 farms.

### 3.4. Associations Between Farm Characteristics and MDR in Enterobacterales in Farm Samples

Associations are shown in Table 5. Significant associations between farm characteristics and MDR included the following: annual pig turnover above 100 (with no MDR found in pigs from low-density pig farms); pig fecal waste burnt on farms rather than dumped, although this only involved three Enterobacterales isolates; pig slurry channelled into pits rather than freely on to farm soil; antimicrobials used for treatment and prophylaxis in the last 12 months.

## 4. Discussion

This first study in Ghana conducted a microbiological assessment of pig farm feed, water, and pig slurry, revealing that over three-quarters of these samples contained Enterobacterales. AMR was common, with both MDR and phenotypic colistin resistance found in about one quarter of bacterial isolates. The findings related to the key objectives are discussed below.

First, regarding farm characteristics and practices, the average annual pig production turnover was 165, and self-made farm feed was the most commonly used type of pig feed. These results are similar to recent studies from pig farms in Accra, which are usually small to medium in size [12,20]. Boreholes served as the main source of farm water, similar to many farms in Accra, where a limited local water supply has led farmers to invest in dug-out boreholes and wells, with 35% and 33% of farms in the Ashanti region of Ghana relying on boreholes and well water, respectively [21].

All farms maintained pig manure, which was used for crop farming on the majority of them. Generally, farms in Ghana do not treat the manure to eliminate bacteria or other harmful substances, potentially leading to the contamination of crops with resistant bacteria, as evidenced by several studies across Ghana [22,23]. While a few farms incinerated their manure, this was typically performed several days after exposure, which could pose an environmental hazard. Additionally, discharging pig slurry onto farm soil may contribute to agricultural runoffs during rainfall and create a source of infection for free-range animals, thereby posing a risk to nearby households and the environment [24].

Few farms had functional footbaths, which can lead to cross-contamination between the indoor pen and outdoor areas. Poor adherence to proper cleaning and disinfection protocols echoes findings from a study across ten European countries [25], which documented inconsistent practices and a lack of in-depth understanding regarding the effects of cross-contamination between different farm sections.

Antibiotics were administered to pigs in 57% of farms, and in half of these cases, farmers, rather than a veterinarian, determined the type and dosage of treatment. This pattern aligns with similar findings reported in Kumasi, Ghana, where Dogbatse et al. observed that 22.4% of farmers independently decided on antibiotic use without veterinary consultation [26]. Nearly half of the farms used antibiotics for prophylaxis. Among those that reported on the disposal of antibiotic containers, either half buried them or discarded them on the farm, practices that constitute a risk for the development of drug-resistant bacteria.

Second, with respect to Enterobacterales and patterns of AMR, MDR, and colistin resistance, over three-quarters of samples contained Enterobacterales, with *E. coli* predominating in pig slurry and *Enterobacter* spp. predominating in farm feed. This finding corroborates studies on farm feed in Ireland [27], Nigeria [28], the USA [29] and Ghana [15], where *Enterobacter* spp. was also the dominant bacterial group with high levels of AMR to ampicillin, tetracycline, and trimethoprim-sulfamethoxazole, as found in our study. High levels of ampicillin resistance amongst *E. coli* were also found in feeds from Colombia and Kenya. [30,31], although resistance levels to tetracycline (10%) and trimethoprim-sulfamethoxazole (7%) were lower in Kenya. Collectively, these findings indicate that animal feed is a potent reservoir of AMR bacteria, which may drive infection and thereby increase the use of antimicrobials in farm animals. The 38.5% MDR prevalence in farm feed, higher than in pig slurry and farm water, suggests contamination from the farm environment or other farm practices.

The prevalence of Enterobacterales in farm water closely aligns with findings from a study conducted by Ahmed and colleagues on drinking water samples in the Greater Accra region, which reported no significant differences in the proportion of resistant Enterobacterales in untreated water (i.e., borehole and well water) and treated water (i.e., tap water) [32]. Similar AMR bacteria have been reported in farm water sources across other regions in Ghana [21,33], indicating widespread environmental contamination and possible seepage of untreated sewage or wastewater into groundwater [34,35]. Pig slurry samples from our farms showed a high prevalence of *E. coli*, with resistance patterns mirroring those reported in other Ghanaian livestock studies [12,36,37], particularly for tetracyclines and trimethoprim-sulfamethoxazole. However, our pig slurry samples showed lower resistance levels compared to studies from Chinese pig farms, where tetracycline resistance exceeded 80%, most likely due to differences in antibiotic use intensity [38].

Phenotypic colistin resistance was found in nearly a quarter of farm samples, similar to a study in Spain, where colistin resistance persisted even on farms with reduced antibiotic use, indicating potential environmental maintenance of resistance genes [39]. Considering colistin’s status as a last-resort antibiotic, the presence of such high levels of resistance is alarming, as colistin-resistant Enterobacterales can be transmitted from pigs to humans. The lower *mcr-1* genotypic prevalence compared to phenotypic resistance suggests that resistance may be driven by other plasmid-borne *mcr* genes (*mcr-2* to *mcr-10*), the presence of chromosomal mutations, or other shared phenotypic mechanisms of resistance to antimicrobials used on these farms [40,41].

Third, regarding factors associated with MDR, no samples from farms with 100 or fewer pigs had MDR, in contrast to the larger farms, where MDR prevalence was 50%. We speculate that higher animal density facilitates rapid pathogen spread and increased infection risk, making farmers more reliant on prophylactic antibiotics rather than on targeted treatment, which may in turn increase selective pressure for the development of drug-resistant bacteria. The increased association of MDR with pig slurry channelled into covered or uncovered pits compared to free-draining systems may be due to anaerobic conditions in these pits that slow antibiotic breakdown, resulting in prolonged exposure to sublethal doses that select for resistant bacteria [42]. The lower prevalence of MDR in free-draining systems might also be attributed to prolonged exposure to sunlight, higher temperatures, and drier conditions, all of which significantly reduce bacterial viability in wastewater and sludge environments [43,44].

Fourth, we found no evidence of antibiotic residues in our feed and farm water samples, which was partly expected since no antibiotics were added to these sample types. However, despite antibiotic use in animals across all farms, pig slurry samples also showed no detectable antimicrobial residues. A recent study in Europe analyzing manure from pig and chicken farms found antibiotic residues more frequently in chicken manure than in pig manure, with 28% of pig manure samples containing residues [45]. The absence of antibiotic residues in our study may be due to various factors, including microbial degradation and chemical breakdown, which can reduce antibiotic residues in pig slurry over time [46], and this may partly explain our findings.

This study had several strengths. By combining microbiological analysis of farm feed, pig slurry, and farm water with a detailed survey of farm practices, we conducted a comprehensive baseline study on AMR dynamics in these samples in the Greater Accra region of Ghana. The study was also conducted and reported in accordance with STROBE guidelines (Strengthening the Reporting of Observational Studies in Epidemiology) [47]. However, a limitation was our inability to detect antibiotic residues, potentially due to the sensitivity limits of our screening assays, which may not have been able to detect low-level residues in complex matrices like pig slurry [46,48]. This limitation was related to costs. Alternative but more expensive methods would include thin-layer chromatography (TCL), ultra-high-performance liquid chromatography, mass and ultraviolet spectrometry, gas chromatography, colorimetry, fluorimetry, conductometry, and the amperometric titration method [49,50].

Despite this limitation, the study findings underscore the need for immediate interventions, including improved hygiene for feed, water, and slurry, enhanced biosecurity measures, and stricter antibiotic stewardship with veterinary oversight. Policy recommendations include expanding surveillance to track resistance genes, enforcing existing regulations for antibiotic use, and continuing farmer education programs on sustainable biosecurity practices. Addressing these issues through integrated One Health approaches is essential to mitigate AMR risks and protect both animal and human health in Ghana’s livestock sector.

## 5. Conclusions

This study, conducted on 14 pig farms in the Greater Accra Region of Ghana, found that over three-quarters of the samples collected from farm feed, pig slurry, and farm water contained Enterobacterales. A significant proportion of these bacteria exhibited resistance to tetracycline, trimethoprim-sulfamethoxazole, and ampicillin. Additionally, MDR was observed in 27% of the isolates, with 24% also showing phenotypic resistance to colistin. The study discusses the reasons behind these high levels of resistance and provides recommendations to address the problem.

## Figures and Tables

**Table 1:** Characteristics and practices of selected pig farms in the Greater Accra Region, Ghana, 2024.

Variables		*n*	(%)
Selected farms		14	
Annual pig production turnover	1–100	6	(42.9)
	101–300	5	(35.7)
	>300	3	(21.4)
Farm feed source	Commercial	1	(7.1)
	Self-made	10	(71.5)
	Self-made and Commercial	3	(21.4)
Antimicrobials added to farm feed	Yes	0	(0)
	No	14	(100)
Animal drinking water source	Boreholes	8	(57.1)
	Tap water	4	(28.6)
	Well water	2	(14.3)
Pig fecal waste management	Waste dumped on farm and collected for manure	7	(50.0)
	Waste just dumped on farm	5	(35.7)
	Waste burnt	2	(14.3)
Pig slurry management	Channelled freely into farm soil	9	(64.3)
	Channelled into covered pit	4	(28.6)
	Channelled into uncovered pit	1	(7.1)
Presence of functional foot baths	Yes	3	(21.4)
	No	11	(78.6)
Use of personal protective equipment	Yes	14	(100)
	No	0	(0)
Antimicrobial treatment of pigs in the last 12 months	Yes	8	(57.1)
	No	6	(42.9)
If yes, veterinarian was consulted before treatment?	Yes	4	(50.0)
	No	4	(50.0)
If no, who decided on type of treatment?	The farmer decided on antimicrobial and dosage	4	(100)
Prophylactic antimicrobials given to pigs in the last 12 months	Yes	6	(42.9)
	No	8	(57.1)
Disposal of used antimicrobial containers	Dumped into a bin	2	(14.3)
	Burnt	4	(28.6)
	Buried	2	(14.3)
	Dumped on the farm	5	(35.7)

Footnote: One farm did not use antibiotics for either treatment or prophylaxis.

**Table 2:** Enterobacterales from farm feed, pig slurry, and farm water on 14 selected pig farms in the Greater Accra region, Ghana, 2024.

Sample Type	Number of Samples	Samples with Enterobacterales		Samples with *E. coli* Only		Samples with *Enterobacter* spp. Only		Samples with Both *E. coli* and *Enterobacter* spp.	
	N	*n*	(%)	*n*	(%)	*n*	(%)	*n*	(%)
Farm Feed	14	12	(85.7)	3	(25.0)	8	(66.7)	1	(8.3)
Pig Slurry	14	11	(78.6)	9	(81.8)	0	(0.0)	2	(18.2)
Farm Water	14	7	(50.0)	3	(42.9)	4	(57.1)	0	(0)
All samples	42	30	(71.4)	15	(50.0)	12	(40.0)	3	(10.0)

Footnote: the 12 farm feeds contaminated with Enterobacterales were self-made (8), commercial (1), and commercial and self-made (3); the 7 farm water sources contaminated with Enterobacterales all came from borehole water.

**Table 3:** Multi-drug resistant Enterobacterales isolated from farm feed, pig slurry, and farm water on 14 selected pig farms in the Greater Accra region of Ghana, 2024.

Samples	Total	MDR		R3	R4	R5
	*n*	*n*	(%)	*n*	*n*	*n*
Farm feed	13	5	(38.5)	3	0	2
Pig slurry	13	3	(23.1)	2	0	1
Farm water	7	1	(14.3)	0	1	0

Footnotes: MDR = multi-drug resistance; R3 = resistance to 3 antimicrobial classes; R4 = resistance to 4 antimicrobial classes; R5 = resistance to 5 antimicrobial classes.

**Table 4:** Colistin resistance of Enterobacterales in farm feed, farm water, and pig slurry of the respective pig farms in the Greater Accra region, 2024.

Variable	Resistance	
Total samples (N = 42)	*n*	(%)
Phenotypic resistance		
*E. coli*	3	(7.1)
*Enterobacter* spp.	7	(16.7)
Molecular resistance *		
*E. coli*	2	(66.7)
*Enterobacter* spp.	1	(14.3)

Footnotes: * Molecular = Detection of the *mcr-1* gene; detection was performed in only isolates that were phenotypically positive for colistin resistance.

**Table 5:** Farm characteristics and practices associated with MDR in Enterobacterales in 14 selected farms in the Greater Accra region, 2024.

Variable	Total	MDR		PR	95% CI	*p* Value
		*n*	(%)			
Total Enterobacterales	33	9	(27.3)			
Annual Pig turnover						
1–100	14	0	(0.0)	1		
101–300	17	8	(47.1)	14	(0.9–222)	<0.01
>300	2	1	(50.0)	15	(0.8–286)	<0.05
Animal drinking water source						
Borehole	22	5	(22.7)	1		
Well water	3	1	(33.3)	1.5	(0.2–8.7)	0.7
Tap water	8	3	(37.5)	1.7	(0.5–5.4)	0.4
Pig fecal waste management						
Waste dumped on farm and collected for manure	16	3	(18.8)	1		
Waste just dumped on farm	14	3	(21.4)	1.1	(0.3–4.8)	0.8
Waste burnt	3	3	(100)	4.7	(1.6–13.8)	<0.05
Pig slurry management						
Channelled freely into farm soil	21	3	(14.3)	1		
Channelled into covered/uncovered pit	12	6	(50.0)	3.5	(1.1–11.5)	<0.05
Presence of functional foot baths						
No	26	7	(26.9)	1		
Yes	7	2	(28.6)	1.1	(0.3–4.8)	0.9
Antimicrobial treatment of pigs in last 12 months						
No	17	2	(11.8)	1		
Yes	16	7	(43.8)	3.7	(0.9–15.3)	0.09
Prophylactic antimicrobials to pigs in last 12 months						
No	23	0	(0.0)	1		
Yes	10	1	(10.0)	6.5	(0.3–148)	0.2
Antimicrobials used for both treatment and prophylaxis in the last 12 months						
No	29	0	(0.0)	1		
Yes	4	1	(25.0)	18	(0.8–382)	<0.05
Disposal of used antimicrobial containers						
Dumped into a bin	8	2	(25.0)	1		
Burnt or buried	12	4	(33.3)	1.3	(0.3–5.6)	0.7
Dumped on the farm	10	3	(30.0)	1.2	(0.3–5.5)	0.8

Footnotes: MDR = multi-drug resistance; PR = prevalence ratio; CI = confidence intervals.

## Data Availability

Requests to access the data from the study should be sent to the corresponding author.
